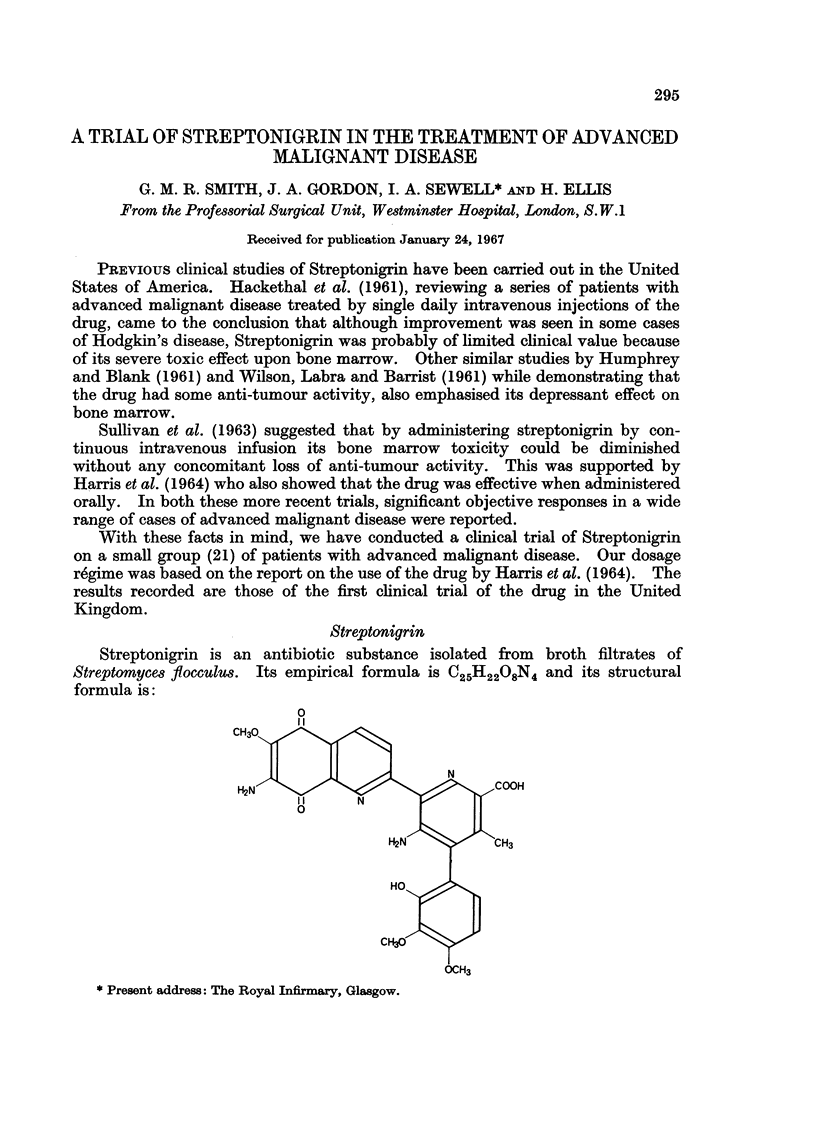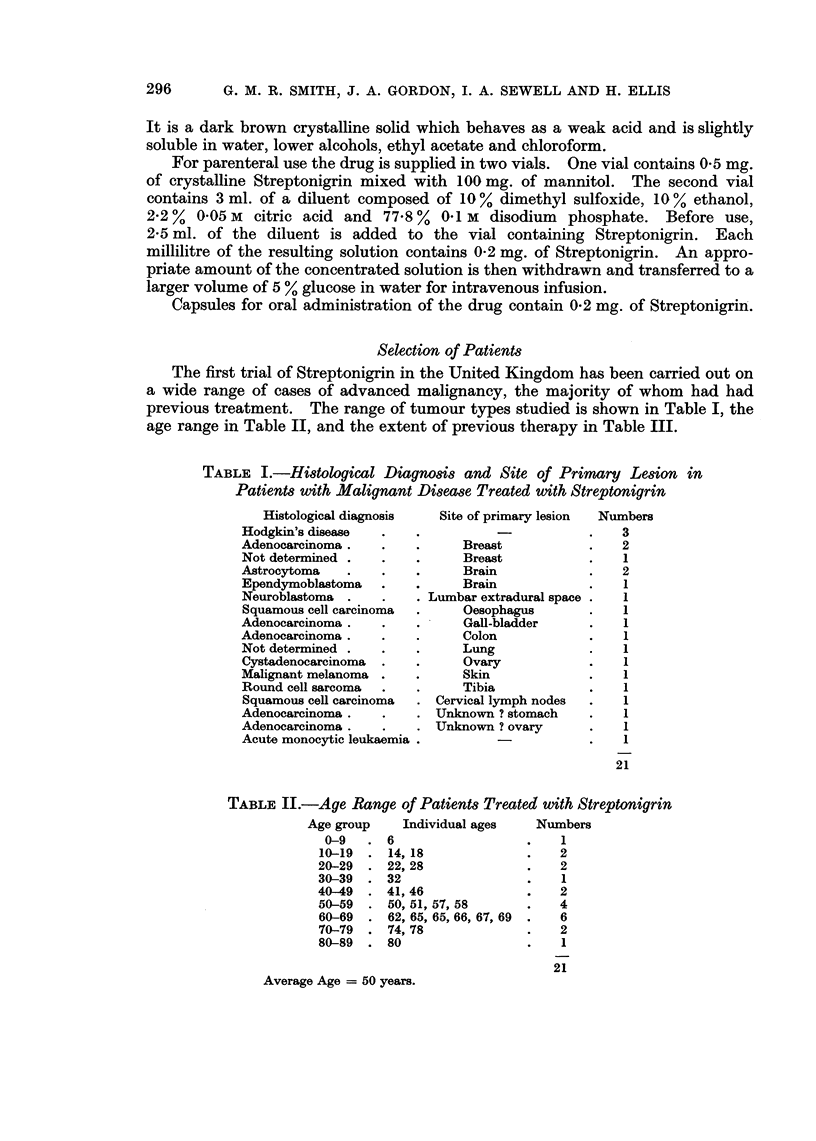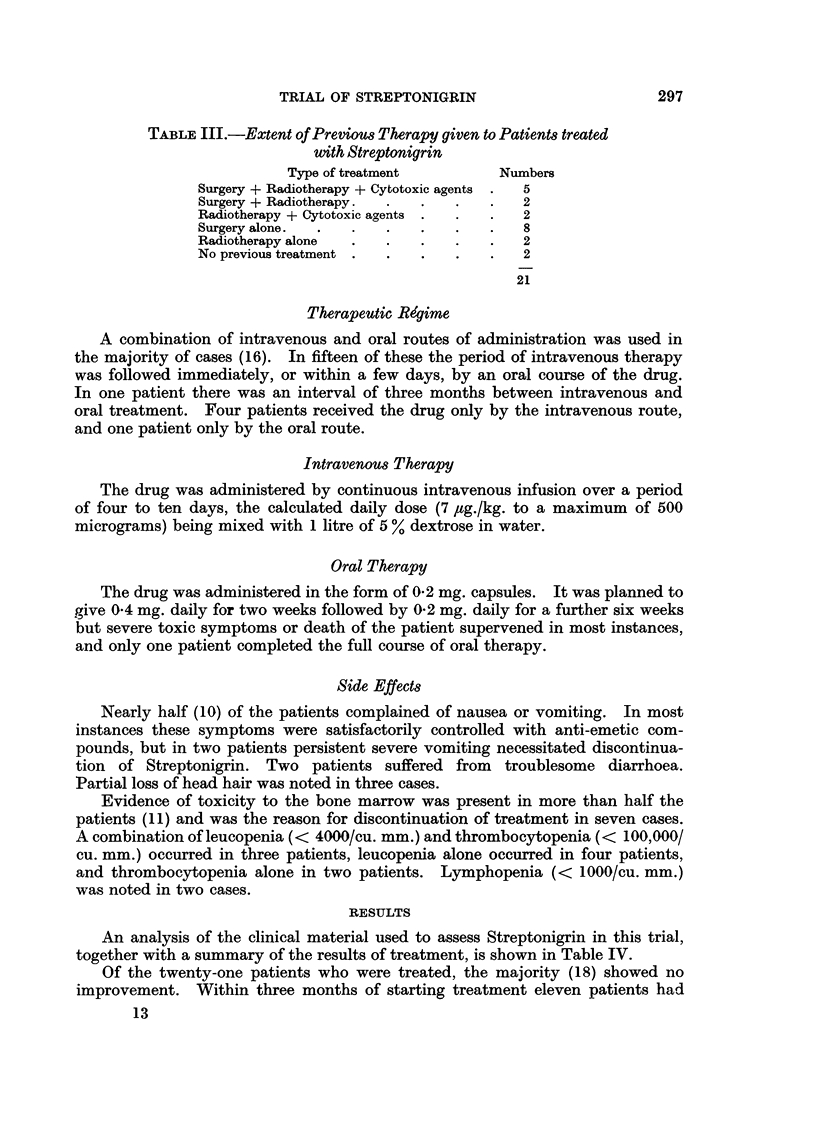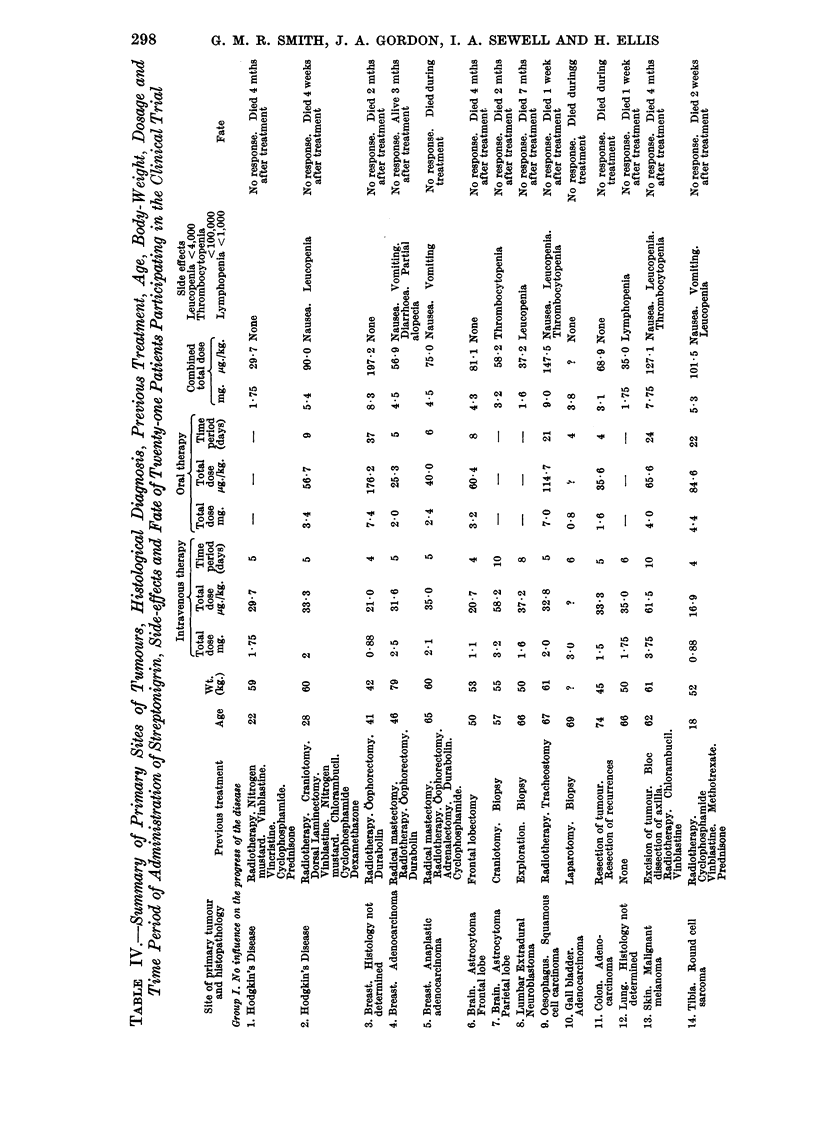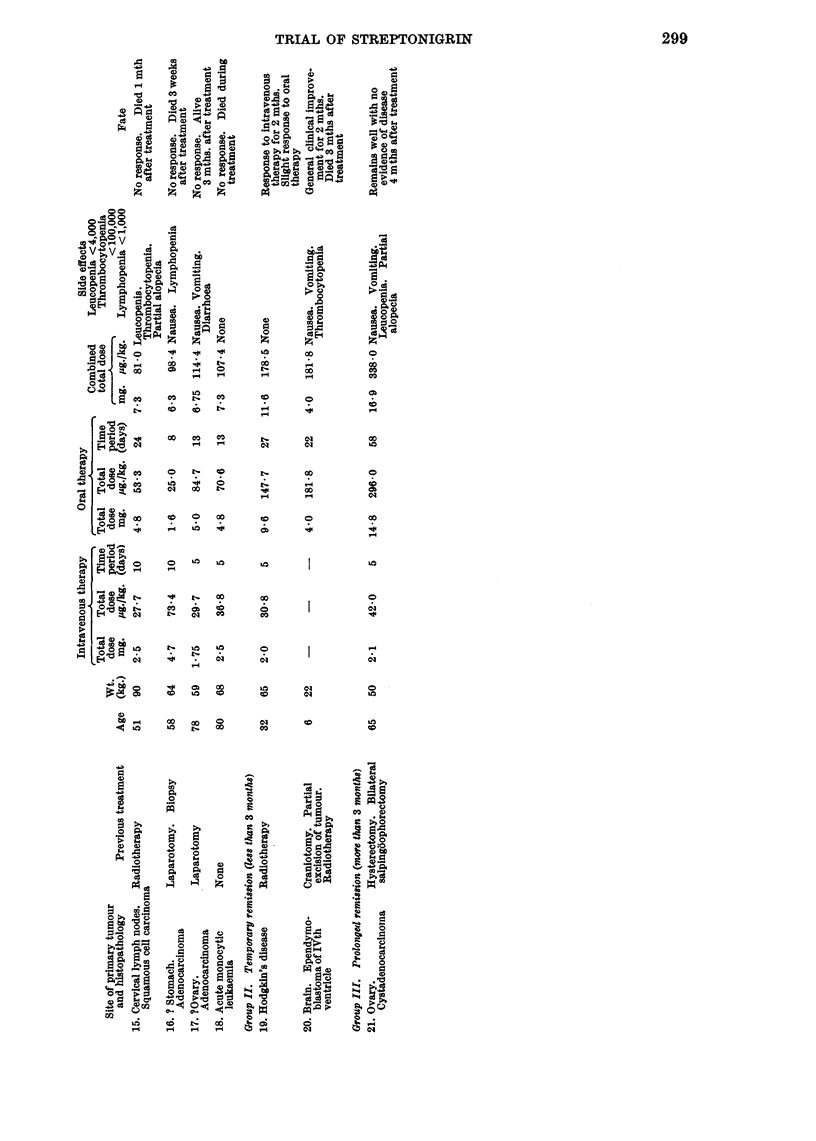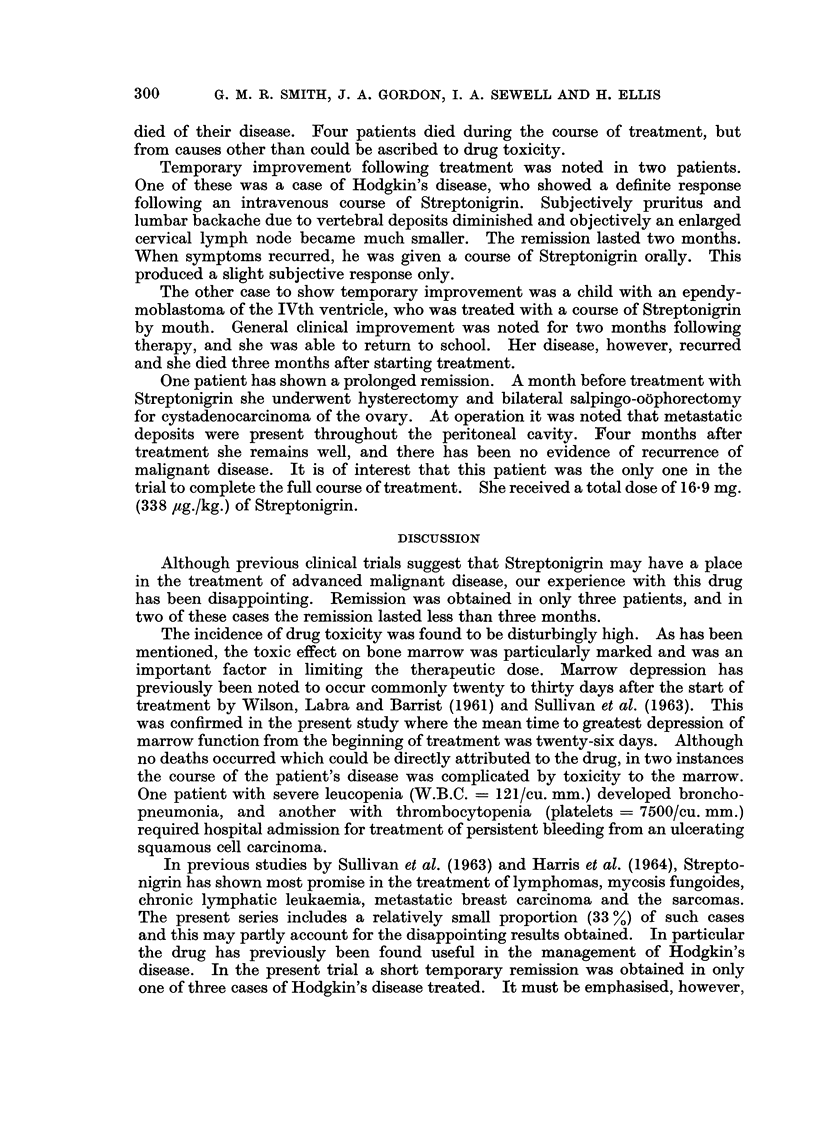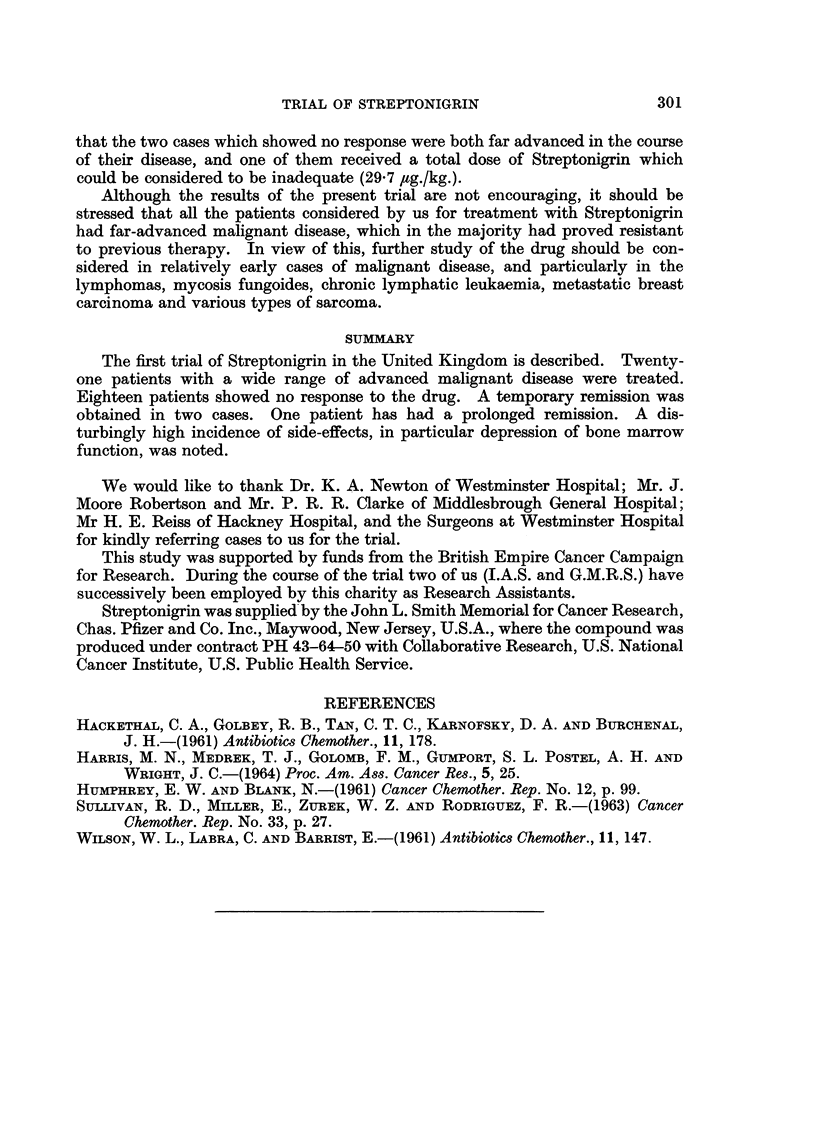# A trial of streptonigrin in the treatment of advanced malignant disease.

**DOI:** 10.1038/bjc.1967.32

**Published:** 1967-06

**Authors:** G. M. Smith, J. A. Gordon, I. A. Sewell, H. Ellis


### A TRIAL OF STREPTONIGRIN IN THE TREATMENT OF ADVANCED MALIGNANT DISEASE

G. M. R. SMITH, J. A. GORDON, I. A. SEWELL* AND H. ELLIS
*From the Professorial Surgical Unit, Westminster Hospital, London, S.W.1*

Received for publication January 24, 1967

PREVIOUS clinical studies of Streptonigrin have been carried out in the United States of America. Hackethal *et al.* (1961), reviewing a series of patients with advanced malignant disease treated by single daily intravenous injections of the drug, came to the conclusion that although improvement was seen in some cases of Hodgkin's disease, Streptonigrin was probably of limited clinical value because of its severe toxic effect upon bone marrow. Other similar studies by Humphrey and Blank (1961) and Wilson, Labra and Barrist (1961) while demonstrating that the drug had some anti-tumour activity, also emphasised its depressant effect on bone marrow.

Sullivan *et al.* (1963) suggested that by administering streptonigrin by continuous intravenous infusion its bone marrow toxicity could be diminished without any concomitant loss of anti-tumour activity. This was supported by Harris *et al.* (1964) who also showed that the drug was effective when administered orally. In both these more recent trials, significant objective responses in a wide range of cases of advanced malignant disease were reported.

With these facts in mind, we have conducted a clinical trial of Streptonigrin on a small group (21) of patients with advanced malignant disease. Our dosage régime was based on the report on the use of the drug by Harris *et al.* (1964). The results recorded are those of the first clinical trial of the drug in the United Kingdom.

*Streptonigrin*

Streptonigrin is an antibiotic substance isolated from broth filtrates of *Streptomyces flocculus*. Its empirical formula is $C_{25}H_{22}O_8N_4$ and its structural formula is:

* Present address: The Royal Infirmary, Glasgow.

It is a dark brown crystalline solid which behaves as a weak acid and is slightly soluble in water, lower alcohols, ethyl acetate and chloroform.

For parenteral use the drug is supplied in two vials. One vial contains 0·5 mg. of crystalline Streptonigrin mixed with 100 mg. of mannitol. The second vial contains 3 ml. of a diluent composed of 10% dimethyl sulfoxide, 10% ethanol, 2·2% 0·05 M citric acid and 77·8% 0·1 M disodium phosphate. Before use, 2·5 ml. of the diluent is added to the vial containing Streptonigrin. Each millilitre of the resulting solution contains 0·2 mg. of Streptonigrin. An appropriate amount of the concentrated solution is then withdrawn and transferred to a larger volume of 5% glucose in water for intravenous infusion.

Capsules for oral administration of the drug contain 0·2 mg. of Streptonigrin.

### *Selection of Patients*

The first trial of Streptonigrin in the United Kingdom has been carried out on a wide range of cases of advanced malignancy, the majority of whom had had previous treatment. The range of tumour types studied is shown in Table I, the age range in Table II, and the extent of previous therapy in Table III.

TABLE I.—*Histological Diagnosis and Site of Primary Lesion in Patients with Malignant Disease Treated with Streptonigrin*

| Histological diagnosis | Site of primary lesion | Numbers |
|---|---|---|
| Hodgkin's disease | — | 3 |
| Adenocarcinoma | Breast | 2 |
| Not determined | Breast | 1 |
| Astrocytoma | Brain | 2 |
| Ependymoblastoma | Brain | 1 |
| Neuroblastoma | Lumbar extradural space | 1 |
| Squamous cell carcinoma | Oesophagus | 1 |
| Adenocarcinoma | Gall-bladder | 1 |
| Adenocarcinoma | Colon | 1 |
| Not determined | Lung | 1 |
| Cystadenocarcinoma | Ovary | 1 |
| Malignant melanoma | Skin | 1 |
| Round cell sarcoma | Tibia | 1 |
| Squamous cell carcinoma | Cervical lymph nodes | 1 |
| Adenocarcinoma | Unknown ? stomach | 1 |
| Adenocarcinoma | Unknown ? ovary | 1 |
| Acute monocytic leukaemia | — | 1 |
| | | 21 |

TABLE II.—*Age Range of Patients Treated with Streptonigrin*

| Age group | Individual ages | Numbers |
|---|---|---|
| 0–9 | 6 | 1 |
| 10–19 | 14, 18 | 2 |
| 20–29 | 22, 28 | 2 |
| 30–39 | 32 | 1 |
| 40–49 | 41, 46 | 2 |
| 50–59 | 50, 51, 57, 58 | 4 |
| 60–69 | 62, 65, 65, 66, 67, 69 | 6 |
| 70–79 | 74, 78 | 2 |
| 80–89 | 80 | 1 |
| | | 21 |

Average Age = 50 years.

Table III.—*Extent of Previous Therapy given to Patients treated with Streptonigrin*

| Type of treatment | Numbers |
|---|---|
| Surgery + Radiotherapy + Cytotoxic agents . | 5 |
| Surgery + Radiotherapy . . . . . | 2 |
| Radiotherapy + Cytotoxic agents . . . | 2 |
| Surgery alone . . . . . . . | 8 |
| Radiotherapy alone . . . . . | 2 |
| No previous treatment . . . . . | 2 |
| | 21 |

*Therapeutic Régime*

A combination of intravenous and oral routes of administration was used in the majority of cases (16). In fifteen of these the period of intravenous therapy was followed immediately, or within a few days, by an oral course of the drug. In one patient there was an interval of three months between intravenous and oral treatment. Four patients received the drug only by the intravenous route, and one patient only by the oral route.

*Intravenous Therapy*

The drug was administered by continuous intravenous infusion over a period of four to ten days, the calculated daily dose (7 $\mu$g./kg. to a maximum of 500 micrograms) being mixed with 1 litre of 5 % dextrose in water.

*Oral Therapy*

The drug was administered in the form of 0·2 mg. capsules. It was planned to give 0·4 mg. daily for two weeks followed by 0·2 mg. daily for a further six weeks but severe toxic symptoms or death of the patient supervened in most instances, and only one patient completed the full course of oral therapy.

*Side Effects*

Nearly half (10) of the patients complained of nausea or vomiting. In most instances these symptoms were satisfactorily controlled with anti-emetic compounds, but in two patients persistent severe vomiting necessitated discontinuation of Streptonigrin. Two patients suffered from troublesome diarrhoea. Partial loss of head hair was noted in three cases.

Evidence of toxicity to the bone marrow was present in more than half the patients (11) and was the reason for discontinuation of treatment in seven cases. A combination of leucopenia ($<$ 4000/cu. mm.) and thrombocytopenia ($<$ 100,000/cu. mm.) occurred in three patients, leucopenia alone occurred in four patients, and thrombocytopenia alone in two patients. Lymphopenia ($<$ 1000/cu. mm.) was noted in two cases.

RESULTS

An analysis of the clinical material used to assess Streptonigrin in this trial, together with a summary of the results of treatment, is shown in Table IV.

Of the twenty-one patients who were treated, the majority (18) showed no improvement. Within three months of starting treatment eleven patients had

TABLE IV.—*Summary of Primary Sites of Tumours, Histological Diagnosis, Previous Treatment, Age, Body-Weight, Dosage and Time Period of Administration of Streptonigrin, Side-effects and Fate of Twenty-one Patients Participating in the Clinical Trial*

| Site of primary tumour and histopathology | Previous treatment | Age | Wt. (kg.) | Intravenous therapy: Total dose mg. | Intravenous therapy: Total dose μg./kg. | Intravenous therapy: Time period (days) | Oral therapy: Total dose mg. | Oral therapy: Total dose μg./kg. | Oral therapy: Time period (days) | Combined total dose: mg. | Combined total dose: μg./kg. | Side effects Leucopenia <4,000 Thrombocytopenia <100,000 Lymphopenia <1,000 | Fate |
|---|---|---|---|---|---|---|---|---|---|---|---|---|---|
| *Group I. No influence on the progress of the disease* | | | | | | | | | | | | | |
| 1. Hodgkin's Disease | Radiotherapy. Nitrogen mustard. Vinblastine. Vincristine. Cyclophosphamide. Prednisone | 22 | 59 | 1·75 | 29·7 | 5 | — | — | — | 1·75 | 29·7 | None | No response. Died 4 mths after treatment |
| 2. Hodgkin's Disease | Radiotherapy. Craniotomy. Dorsal Laminectomy. Vinblastine. Nitrogen mustard. Chlorambucil. Cyclophosphamide Dexamethazone | 28 | 60 | 2 | 33·3 | 5 | 3·4 | 56·7 | 9 | 5·4 | 90·0 | Nausea. Leucopenia | No response. Died 4 weeks after treatment |
| 3. Breast. Histology not determined | Radiotherapy. Öophorectomy. Durabolin | 41 | 42 | 0·88 | 21·0 | 4 | 7·4 | 176·2 | 37 | 8·3 | 197·2 | None | No response. Died 2 mths after treatment |
| 4. Breast. Adenocarcinoma | Radical mastectomy. Radiotherapy. Öophorectomy. Durabolin | 46 | 79 | 2·5 | 31·6 | 5 | 2·0 | 25·3 | 5 | 4·5 | 56·9 | Nausea. Vomiting. Diarrhoea. Partial alopecia | No response. Alive 3 mths after treatment |
| 5. Breast. Anaplastic adenocarcinoma | Radical mastectomy. Radiotherapy. Öophorectomy. Adrenalectomy. Durabolin. Cyclophosphamide. | 65 | 60 | 2·1 | 35·0 | 5 | 2·4 | 40·0 | 6 | 4·5 | 75·0 | Nausea. Vomiting | No response. Died during treatment |
| 6. Brain. Astrocytoma Frontal lobe | Frontal lobectomy | 50 | 53 | 1·1 | 20·7 | 4 | 3·2 | 60·4 | 8 | 4·3 | 81·1 | None | No response. Died 4 mths after treatment |
| 7. Brain. Astrocytoma Parietal lobe | Craniotomy. Biopsy | 57 | 55 | 3·2 | 58·2 | 10 | — | — | — | 3·2 | 58·2 | Thrombocytopenia | No response. Died 2 mths after treatment |
| 8. Lumbar Extradural Neuroblastoma | Exploration. Biopsy | 66 | 50 | 1·6 | 37·2 | 8 | — | — | — | 1·6 | 37·2 | Leucopenia | No response. Died 7 mths after treatment |
| 9. Oesophagus. Squamous cell carcinoma | Radiotherapy. Tracheostomy | 67 | 61 | 2·0 | 32·8 | 5 | 7·0 | 114·7 | 21 | 9·0 | 147·5 | Nausea. Leucopenia. Thrombocytopenia | No response. Died 1 week after treatment |
| 10. Gall bladder. Adenocarcinoma | Laparotomy. Biopsy | 69 | ? | 3·0 | ? | 6 | 0·8 | ? | 4 | 3·8 | ? | None | No response. Died duringg treatment |
| 11. Colon. Adeno-carcinoma | Resection of tumour. Resection of recurrences | 74 | 45 | 1·5 | 33·3 | 5 | 1·6 | 35·6 | 4 | 3·1 | 68·9 | None | No response. Died during treatment |
| 12. Lung. Histology not determined | None | 66 | 50 | 1·75 | 35·0 | 6 | — | — | — | 1·75 | 35·0 | Lymphopenia | No response. Died 1 week after treatment |
| 13. Skin. Malignant melanoma | Excision of tumour. Bloc dissection of axilla. Radiotherapy. Chlorambucil. Vinblastine | 62 | 61 | 3·75 | 61·5 | 10 | 4·0 | 65·6 | 24 | 7·75 | 127·1 | Nausea. Leucopenia. Thrombocytopenia | No response. Died 4 mths after treatment |
| 14. Tibia. Round cell sarcoma | Radiotherapy. Cyclophosphamide Vinblastine. Methotrexate. Prednisone | 18 | 52 | 0·88 | 16·9 | 4 | 4·4 | 84·6 | 22 | 5·3 | 101·5 | Nausea. Vomiting. Leucopenia | No response. Died 2 weeks after treatment |

| Site of primary tumour and histopathology | Previous treatment | Age | Wt. (kg.) | Intravenous therapy: Total dose mg. | Intravenous therapy: Total dose µg./kg. | Intravenous therapy: Time period (days) | Oral therapy: Total dose mg. | Oral therapy: Total dose µg./kg. | Oral therapy: Time period (days) | Combined total dose: mg. | Combined total dose: µg./kg. | Side effects Leucopenia <4,000 Thrombocytopenia <100,000 Lymphopenia <1,000 | Fate |
|---|---|---|---|---|---|---|---|---|---|---|---|---|---|
| 15. Cervical lymph nodes. Squamous cell carcinoma | Radiotherapy | 51 | 90 | 2·5 | 27·7 | 10 | 4·8 | 53·3 | 24 | 7·3 | 81·0 | Leucopenia. Thrombocytopenia. Partial alopecia | No response. Died 1 mth after treatment |
| 16. ? Stomach. Adenocarcinoma | Laparotomy. Biopsy | 58 | 64 | 4·7 | 73·4 | 10 | 1·6 | 25·0 | 8 | 6·3 | 98·4 | Nausea. Lymphopenia | No response. Died 3 weeks after treatment |
| 17. ?Ovary. Adenocarcinoma | Laparotomy | 78 | 59 | 1·75 | 29·7 | 5 | 5·0 | 84·7 | 13 | 6·75 | 114·4 | Nausea. Vomiting. Diarrhoea | No response. Alive 3 mths. after treatment |
| 18. Acute monocytic leukaemia | None | 80 | 68 | 2·5 | 36·8 | 5 | 4·8 | 70·6 | 13 | 7·3 | 107·4 | None | No response. Died during treatment |
| *Group II. Temporary remission (less than 3 months)* | | | | | | | | | | | | | |
| 19. Hodgkin's disease | Radiotherapy | 32 | 65 | 2·0 | 30·8 | 5 | 9·6 | 147·7 | 27 | 11·6 | 178·5 | None | Response to intravenous therapy for 2 mths. Slight response to oral therapy |
| 20. Brain. Ependymoblastoma of IVth ventricle | Craniotomy. Partial excision of tumour. Radiotherapy | 6 | 22 | — | — | — | 4·0 | 181·8 | 22 | 4·0 | 181·8 | Nausea. Vomiting. Thrombocytopenia | General clinical improvement for 2 mths. Died 3 mths after treatment |
| *Group III. Prolonged remission (more than 3 months)* | | | | | | | | | | | | | |
| 21. Ovary. Cystadenocarcinoma | Hysterectomy. Bilateral salpingöophorectomy | 65 | 50 | 2·1 | 42·0 | 5 | 14·8 | 296·0 | 58 | 16·9 | 338·0 | Nausea. Vomiting. Leucopenia. Partial alopecia | Remains well with no evidence of disease 4 mths after treatment |

died of their disease. Four patients died during the course of treatment, but from causes other than could be ascribed to drug toxicity.

Temporary improvement following treatment was noted in two patients. One of these was a case of Hodgkin's disease, who showed a definite response following an intravenous course of Streptonigrin. Subjectively pruritus and lumbar backache due to vertebral deposits diminished and objectively an enlarged cervical lymph node became much smaller. The remission lasted two months. When symptoms recurred, he was given a course of Streptonigrin orally. This produced a slight subjective response only.

The other case to show temporary improvement was a child with an ependymoblastoma of the IVth ventricle, who was treated with a course of Streptonigrin by mouth. General clinical improvement was noted for two months following therapy, and she was able to return to school. Her disease, however, recurred and she died three months after starting treatment.

One patient has shown a prolonged remission. A month before treatment with Streptonigrin she underwent hysterectomy and bilateral salpingo-oöphorectomy for cystadenocarcinoma of the ovary. At operation it was noted that metastatic deposits were present throughout the peritoneal cavity. Four months after treatment she remains well, and there has been no evidence of recurrence of malignant disease. It is of interest that this patient was the only one in the trial to complete the full course of treatment. She received a total dose of 16·9 mg. (338 $\mu$g./kg.) of Streptonigrin.

### DISCUSSION

Although previous clinical trials suggest that Streptonigrin may have a place in the treatment of advanced malignant disease, our experience with this drug has been disappointing. Remission was obtained in only three patients, and in two of these cases the remission lasted less than three months.

The incidence of drug toxicity was found to be disturbingly high. As has been mentioned, the toxic effect on bone marrow was particularly marked and was an important factor in limiting the therapeutic dose. Marrow depression has previously been noted to occur commonly twenty to thirty days after the start of treatment by Wilson, Labra and Barrist (1961) and Sullivan *et al.* (1963). This was confirmed in the present study where the mean time to greatest depression of marrow function from the beginning of treatment was twenty-six days. Although no deaths occurred which could be directly attributed to the drug, in two instances the course of the patient's disease was complicated by toxicity to the marrow. One patient with severe leucopenia (W.B.C. = 121/cu. mm.) developed bronchopneumonia, and another with thrombocytopenia (platelets = 7500/cu. mm.) required hospital admission for treatment of persistent bleeding from an ulcerating squamous cell carcinoma.

In previous studies by Sullivan *et al.* (1963) and Harris *et al.* (1964), Streptonigrin has shown most promise in the treatment of lymphomas, mycosis fungoides, chronic lymphatic leukaemia, metastatic breast carcinoma and the sarcomas. The present series includes a relatively small proportion (33 %) of such cases and this may partly account for the disappointing results obtained. In particular the drug has previously been found useful in the management of Hodgkin's disease. In the present trial a short temporary remission was obtained in only one of three cases of Hodgkin's disease treated. It must be emphasised, however, that the two cases which showed no response were both far advanced in the course of their disease, and one of them received a total dose of Streptonigrin which could be considered to be inadequate (29·7 $\mu$g./kg.).

Although the results of the present trial are not encouraging, it should be stressed that all the patients considered by us for treatment with Streptonigrin had far-advanced malignant disease, which in the majority had proved resistant to previous therapy. In view of this, further study of the drug should be considered in relatively early cases of malignant disease, and particularly in the lymphomas, mycosis fungoides, chronic lymphatic leukaemia, metastatic breast carcinoma and various types of sarcoma.

SUMMARY

The first trial of Streptonigrin in the United Kingdom is described. Twenty-one patients with a wide range of advanced malignant disease were treated. Eighteen patients showed no response to the drug. A temporary remission was obtained in two cases. One patient has had a prolonged remission. A disturbingly high incidence of side-effects, in particular depression of bone marrow function, was noted.

We would like to thank Dr. K. A. Newton of Westminster Hospital; Mr. J. Moore Robertson and Mr. P. R. R. Clarke of Middlesbrough General Hospital; Mr H. E. Reiss of Hackney Hospital, and the Surgeons at Westminster Hospital for kindly referring cases to us for the trial.

This study was supported by funds from the British Empire Cancer Campaign for Research. During the course of the trial two of us (I.A.S. and G.M.R.S.) have successively been employed by this charity as Research Assistants.

Streptonigrin was supplied by the John L. Smith Memorial for Cancer Research, Chas. Pfizer and Co. Inc., Maywood, New Jersey, U.S.A., where the compound was produced under contract PH 43–64–50 with Collaborative Research, U.S. National Cancer Institute, U.S. Public Health Service.

### REFERENCES

Hackethal, C. A., Golbey, R. B., Tan, C. T. C., Karnofsky, D. A. and Burchenal, J. H.—(1961) *Antibiotics Chemother.*, **11**, 178.

Harris, M. N., Medrek, T. J., Golomb, F. M., Gumport, S. L. Postel, A. H. and Wright, J. C.—(1964) *Proc. Am. Ass. Cancer Res.*, **5**, 25.

Humphrey, E. W. and Blank, N.—(1961) *Cancer Chemother. Rep.* No. 12, p. 99.

Sullivan, R. D., Miller, E., Zurek, W. Z. and Rodriguez, F. R.—(1963) *Cancer Chemother. Rep.* No. 33, p. 27.

Wilson, W. L., Labra, C. and Barrist, E.—(1961) *Antibiotics Chemother.*, **11**, 147.